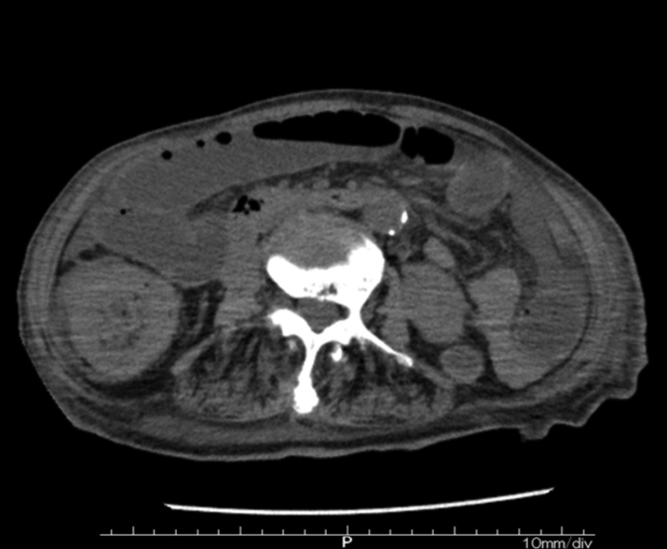# Stent Placement Using a Combination of Decompression Tube and Colonoscopy for Malignant Ileocolonic Obstruction

**DOI:** 10.1016/j.gastha.2023.04.003

**Published:** 2023-04-15

**Authors:** Mie Tanabe, Naoki Ishii, Daisuke Inagaki

**Affiliations:** 1 Department of Surgery, Tokyo Shinagawa Hospital, Tokyo, Japan; 2 Gastroenterology Division, Tokyo Shinagawa Hospital, Tokyo, Japan

The placement of self-expandable metallic stents (SEMSs) from colon to terminal ileum for malignant ileocolonic obstruction (MIO) is technically challenging, often necessitating surgery. However, it is recommended for patients with severe comorbidities, who cannot tolerate general anesthesia and surgery. We performed the first case of SEMS placement for MIO using the combination of a decompression tube and colonoscopy.

A 101-year-old female underwent computed tomography for the assessment of abdominal pain and vomiting. MIO was suspected (Figure A1). A decompression tube was first inserted. A colonoscopy without bowel purge demonstrated a protruding-type colonic cancer occupying the ascending colon (Figure A). Due to advanced age and comorbidities, SEMS placement was planned to alleviate the symptoms. Due to ileocolonic anatomy and difficulties in identifying the opening mouth for colonoscopic guidewire insertion, the guidewire was, instead, passed into the ascending colon through a decompression tube, grasped by colonoscopy forceps and brought out from the working channel of the colonoscope (Figure B). The SEMS was deployed using the guidewire from the ascending colon into the terminal ileum (Figure C and D). The decompression tube was pulled away. After the -procedure, the patient had no episodes of abdominal pain or vomiting and was able to eat after stenting.

## Figures and Tables

**Figure A1:**